# High Prevalence of Nutritional Risk Among Pulmonary Patients Living on the Tibetan Plateau

**DOI:** 10.3389/fnut.2022.872457

**Published:** 2022-05-10

**Authors:** Chilie Quncuo, Ying Liang, Qiuyu Li, Xiaoli She, Bian Ma Cuo, Bianba Qiongda, Meilang ChuTso, Yongchang Sun

**Affiliations:** ^1^Department of Respiratory and Critical Care Medicine, Tibet Autonomous Region People's Hospital, Lhasa, China; ^2^Department of Respiratory and Critical Care Medicine, Peking University Third Hospital, Beijing, China

**Keywords:** nutritional risk, NRS-2002, high altitude, respiratory diseases, pulmonology inpatients

## Abstract

**Background:**

Nutritional risk is associated with adverse clinical outcomes and is more prevalent among pulmonology patients than among patients in other departments. High-altitude environments can affect patients with chronic respiratory diseases, but evidence of the prevalence of nutritional risk among hospitalized patients with respiratory diseases in high-altitude areas is limited. This study aimed to investigate the nutritional risk and status of inpatients with different major respiratory diagnoses permanently living on the Tibetan Plateau (≥3,000 m above sea level).

**Methods:**

In this cross-sectional study, we consecutively recruited inpatients admitted to the Department of Respiratory and Critical Care Medicine at the Tibet Autonomous Region People's Hospital of Lhasa between November 2020 and May 2021. We used the Nutrition Risk Screening (NRS) 2002 tool to assess nutritional risk among these patients. An NRS 2002 score ≥3 points indicates nutritional risk; a score ≥5 indicates high nutritional risk. According to NRS-2002 scores, patients were divided into three groups (NRS-2002 0–2, 3–4, and ≥5). The differences in age, sex, major respiratory diagnoses, comorbidities, body mass index, and laboratory findings among the groups were analyzed.

**Results:**

A total of 289 eligible Tibetan patients were enrolled in the study, and 46.1% (133/246) of them were at nutritional risk (NRS-2002 score ≥3). Twenty-one (7.3%) patients were at high nutritional risk (NRS-2002 score ≥5). The proportions of patients at nutritional risk were relatively high among patients with lung cancer (58.8%), interstitial lung disease (58.3%), pulmonary embolism (52.9%), and tuberculosis (50.0%). Laboratory findings showed that patients with NRS-2002 scores of 3–4 and ≥5 had lower red blood cell counts, serum albumin and hemoglobin levels, and higher C-reactive protein (CRP) levels than those with NRS-2002 scores < 3.

**Conclusion:**

The prevalence of nutritional risk was high among pulmonology department inpatients permanently living on the Tibetan Plateau. Patients with lung cancer, interstitial lung disease, pulmonary embolism or tuberculosis were more likely to have nutritional risk than patients with other diagnoses. The nutritional risk of inpatients in the respiratory department in the plateau area should not be ignored, and patients at high nutritional risk should receive timely intervention.

## Introduction

A good nutritional status is associated with a faster recovery from illnesses (1). Therefore, recognition of malnutrition and initiation of adequate nutritional therapy among hospitalized patients are important components of inpatient care. Malnutrition is common among hospitalized patients, with a prevalence ranging from 30 to 50% (2–4). While malnutrition is defined as deficiencies, excesses or imbalances in a person's intake of energy and/or nutrients, the assessment of nutritional risk is mainly performed by evaluations of baseline nutritional status and disease severity (5). It has been widely demonstrated that nutritional risk is closely associated with adverse clinical outcomes (6–8), such as increased morbidity and mortality, prolonging hospital stay and increased healthcare costs.

Nutritional risk is especially prevalent among pulmonology inpatients. A systematic review showed that the prevalence of nutritional risk was higher among pulmonology department patients than among patients in other departments (9). A multicenter study conducted in China also showed that hospitalized patients in the department of pulmonology had a higher prevalence of malnutrition and nutritional risk than those in the departments of gastroenterology, neurology, nephrology, general surgery and thoracic surgery (10). Other studies showed that among hospitalized patients in the department of pulmonology, those with tuberculosis, lung cancer, and chronic obstructive pulmonary disease (COPD) complicated with respiratory failure were more likely to have nutritional risk, with a prevalence ranging from 55 to 65% (11–13).

The Tibetan Plateau is the highest region on Earth, with an average altitude of 4,000 meters above sea level (14). There are relatively few vegetables and fruits in the daily diet, and there is a single diet structure. The food crops are mainly wheat and highland barley, and the meat products are mainly yak meat, mutton and dairy products (15). Although the prevalence of malnutrition in Tibetan children and adolescents was significantly reduced during the past decade (16), the prevalence of malnutrition in the elderly generation of these children and adolescents was still high, which can harm their condition once they are hospitalized due to various diseases. Otherwise, Tibetan adolescents and adults had significantly larger forced vital capacity (FVC) and forced expiratory volume at 1 second (FEV1) than Han individuals who were born and raised at high altitudes, indicating a pattern of adaptation to hypoxia at high altitudes among Tibetan individuals (17). Due to different mechanisms, respiratory diseases can cause hypoxemia, including alveolar hypoventilation, decreased diffusion function, or ventilation-perfusion imbalance. Additionally, a high-altitude environment will aggravate hypoxemia. Therefore, we hypothesized that the nutritional status of patients with respiratory diseases who permanently live on the Tibetan Plateau might be impacted both by illnesses and the environment, and many of them may have a high risk of malnutrition.

However, to our knowledge, there is limited evidence of the nutritional status and nutritional risk of hospitalized patients with respiratory diseases living in high-altitude areas, such as the Tibetan Plateau. Investigating the nutritional risk of hospitalized patients with respiratory diseases can potentially be useful for improving nutritional therapy or other aspects of inpatient care. In this study, we aimed to assess the prevalence of nutritional risk and nutritional status in a group of Tibetan patients with different major respiratory diagnoses who were hospitalized on the Tibetan Plateau.

## Methods

### Study Population and Design

We conducted an observational cross-sectional study in the Department of Respiratory and Critical Care Medicine at the Tibet Autonomous Region People's Hospital, located in the city of Lhasa (~3,650 m above sea level). We consecutively enrolled all inpatients admitted to the department from December 2020 to March 2021.

The inclusion criteria were as follows: (I) inpatients's major diagnoses were respiratory diseases and (II) age ≥18 years. The exclusion criteria were as follows: (1) length of hospitalization <1 day; (2) inability to provide the information about weight loss or food intake necessary to complete the nutritional risk screening due to cognitive dysfunction; (3) inability to accurately measure height and weight due to critical illness; or (4) refusal to participate in this study.

The study protocol was approved by the Ethics Committee of Tibet Autonomous Region People's Hospital (No. ME-TBHP-21-KJ-056). If a patient was admitted to the Department of Respiratory and Critical Care Medicine more than once, data were collected only at the first hospitalization.

### Collection of Clinical Data

Demographic data, including age, sex, height, and weight, were collected. Major respiratory diagnoses included acute exacerbation of chronic obstructive pulmonary disease (AECOPD), community-acquired pneumonia (CAP), pulmonary embolism (PE), pulmonary tuberculosis or tuberculous pleurisy (TB), lung cancer, interstitial lung disease (ILD), asthma, and other respiratory diseases. The clinical diagnoses of all the above respiratory diseases were based on the clinical guidelines of the respective diseases (Supplementary Table 1). Information on other chronic comorbidities was recorded.

### Laboratory Examinations

Routine blood tests and blood biochemistry examinations were performed within the first 24 h after hospitalization. Data were collected on red blood cell counts and hemoglobin (HGB), serum albumin, C-reactive protein (CRP), bilirubin, urea nitrogen, and creatinine levels. For routine blood tests, XN red blood cells and various parameters were calculated by the impedance method from histogram information. Measurement of HGB involved the sodium lauryl sulfate (SLS) hemoglobin method, and the instrument was an XN-10. Biochemical parameters and CRP were quantified using a chemiluminescence method, which was the standard analytical method. Each specific parameter was assessed on a c16000 instrument.

### Assessment of Nutritional Risk

The Nutrition Risk Screening 2002 (NRS-2002) tool was used to assess the nutritional risk of the patients (Supplementary Table 2). This scoring system includes measurements of current potential undernutrition, disease severity and age, which were described previously (18). The chief attending physician and the nurse in charge of the ward jointly completed the NRS-2002 score within 24 h after a patient was admitted to the ward. According to the NRS-2002 scores, patients were divided into three groups (NRS-2002 scores 0–2, 3–4, and ≥5). Patients at “nutritional risk” were defined as those with an NRS-2002 score ≥3, and those at “high nutritional risk” were defined as those with an NRS-2002 score ≥5.

### Statistical Analysis

Data were analyzed using SPSS 23.0 software (IBM, New York, USA). Continuous variables are expressed as the means ± standard deviations. Categorical variables are expressed as frequencies and proportions. One-way ANOVA was used to evaluate differences among means across categories of nutritional risk. The chi-square test or Fisher's exact test was used for categorical variables. The results were considered statistically significant at a *P* value of <0.05.

## Results

### Demographic and Clinical Characteristics of the Participants

During the study period, 350 patients were admitted to the Respiratory Department of Tibet Autonomous Region People's Hospital. The patients were Tibetan residents living on the Tibetan Plateau since their childhood. They came from seven districts in the Tibet Autonomous Region of the People's Republic of China: Lhasa (~3,650 m), Qamdo (~3,500 m), Linzhi (~3,000 m), Shannan (~3,700 m), Nakchu (~5,200 m), Shigatse (~3,900 m) and Ali (~4,500 m). Tibet Autonomous Region People's Hospital is located in the city of Lhasa (~3,650 m above sea level), as described in the previous literature. Sixty-one patients were excluded from the study due to missing necessary medical record data. Ultimately, 289 patients were enrolled in the analysis, consisting of 166 males and 123 females. The mean age was 55.7 ± 17.3 years, and patients ranged from 14 to 96 years of age. The distribution of different major respiratory diagnoses is shown in Table 1.

**Table 1:** Distribution of different major respiratory diagnoses among patients included in the study (*n* = 289).

**Major respiratory diagnosis**	***N* (%)**	**Male**	**Female**
CAP	97 (33.6)	63 (38.0)	34 (27.6)
Asthma	5 (1.7)	2 (1.2)	3 (2.4)
COPD	32 (11.1)	17 (10.2)	15 (12.2)
TB	34 (11.8)	19 (11.4)	15 (12.2)
Lung cancer	34 (11.8)	19 (11.4)	15 (12.2)
PE	17 (5.9)	8 (4.8)	9 (7.3)
ILD	12 (4.2)	6 (3.6)	6 (4.9)
Bronchiectasis	8 (2.8)	3 (1.8)	5 (4.1)
Other	50 (17.3)	29 (17.5)	21 (17.1)

*Data are presented as n (%)*.

*CAP, community-acquired pneumonia; COPD, chronic obstructive pulmonary disease; TB, tuberculosis or tuberculous pleurisy; PE, pulmonary embolism; ILD, interstitial lung disease*.

### Demographic and Clinical Characteristics of the Patients by NRS-2002 Score

As shown in Table 2, patients with an NRS-2002 score < 3 were significantly younger and more likely to be female than those with NRS-2002 scores of 3–4 and ≥5 (53.4 ± 17.4 vs. 55.9 ± 16.8 vs. 71.1 ± 9.2 *P* < 0.001). The proportion of patients aged ≥ 70 years was significantly the highest among those with an NRS-2002 score ≥ 5 (19.2% vs. 21.4% vs. 81% *P* < 0.001), and the prevalence rates of coronary heart disease, heart failure, diabetes and cerebrovascular disease were also highest in this group. Compared to patients with an NRS-2002 score < 3, those with NRS-2002 scores of 3–4 or ≥5 had lower serum albumin levels, lower hemoglobin levels, lower red blood cell counts and higher CRP levels.

**Table 2:** Clinical characteristics by NRS-2002 score.

**Characteristics**	**Overall (*n* = 289)**	**NRS-2002 <3 (*n* = 156)**	**NRS-2002 3–4 (*n* = 112)**	**NRS-2002 ≥5 (*n* = 21)**	***P*-value**
Female sex, *n* (%)	123 (42.6)	74 (47.4)	44 (39.2)	5 (23.8)	<0.001
Age (year)	55.7 ± 17.3	53.4 ± 17.4^b^	55.9 ± 16.8	71.1 ± 9.2	<0.001
Age ≥70 y, *n* (%)	71 (24.6)	30 (19.2)	24 (21.4)	17 (81.0)	<0.001
BMI (kg/m^2^)	24.0 ± 4.8	24.7 ± 5.0	23.3 ± 4.6	22.6 ± 4.2	<0.001
**Comorbidities**, ***n*** **(%)**					
Coronary heart disease	6 (2.1)	3 (1.9)	1 (0.9)	2 (9.5)	0.038
Hypertension	91 (31.5)	43 (27.6)	38 (33.9)	10 (47.6)	0.138
Arrhythmia	15 (5.2)	5 (3.2)	8 (7.1)	2 (9.5)	0.232
Heart failure	20 (6.9)	5 (3.2)	12 (10.7)	3 (14.3)	0.022
Diabetes mellitus	28 (9.7)	8 (5.1)	13 (11.6)	7 (33.3)	<0.001
Hyperlipoidemia	13 (4.5)	6 (3.8)	7 (6.3)	0 (0.0)	0.378
Hematological disease	29 (10)	11 (7.1)	16 (14.3)	2 (9.5)	0.151
Chronic liver disease	41 (14.2)	19 (12.2)	20 (17.9)	2 (9.5)	0.345
Chronic kidney disease	11 (3.8)	4 (2.6)	6 (5.4)	1 (4.8)	0.485
Cerebrovascular disease	11 (3.8)	2 (1.3)	6 (5.4)	3 (14.3)	0.008
**Laboratory findings (Mean** **±SD)**					
RBCs (×10^12^/L)	5.1 ± 1.0	5.3 ± 1.0 ^a^, ^b^	4.8 ± 1.0	4.6 ± 0.9	<0.001
Hemoglobin (g/L)	144.5 ± 29.4	152.8 ± 26.9^a^, ^b^	135.6 ± 30.5	129.7 ± 20.6	<0.001
Albumin (g/L)	35.5 ± 5.2	39.1 ± 3.3^a^, ^b^	31.3 ± 3.5	31.1 ± 4.2	<0.001
Creatinine (μmol/L)	65.2 ± 21.4	64.1 ± 14.1	66.4 ± 26.5	71.3 ± 32.7	0.293
BUN (mmol/L)	4.5 ± 1.8	4.4 ± 1.4	4.7 ± 2.2	4.6 ± 1.7	0.549
Total bilirubin (μmol/L)	13.5 (9.3, 18.2)	14.2 (9.7, 18.4)	12.1 (8.9, 18.1)	14.2 (10.4, 20.5)	0.497
CRP (mg/dL)	8.09 (2.7, 30.6)	4.2 (1.7, 14.4)^a^	18.4 (5.0,55.5)	15.4 (6.2, 77.1)	<0.001

*Data are presented as the means ± standard deviations (SDs) or n (%). Analysis of variance was applied to continuous variables, and the chi-square test was applied to categorical variables, which was described in the methodology*.

a *P < 0.001 compared with NRS-2002 scores 3–4*.

b *P < 0.001 compared with NRS-2002 scores ≥ 5*.

*BMI, body mass index; RBC, red blood cell; BUN, blood urea nitrogen; NRS-2002, Nutrition Risk Screening 2002*.

### Nutritional Risk of Participants With Different Major Respiratory Diagnoses

**Figure 1:**
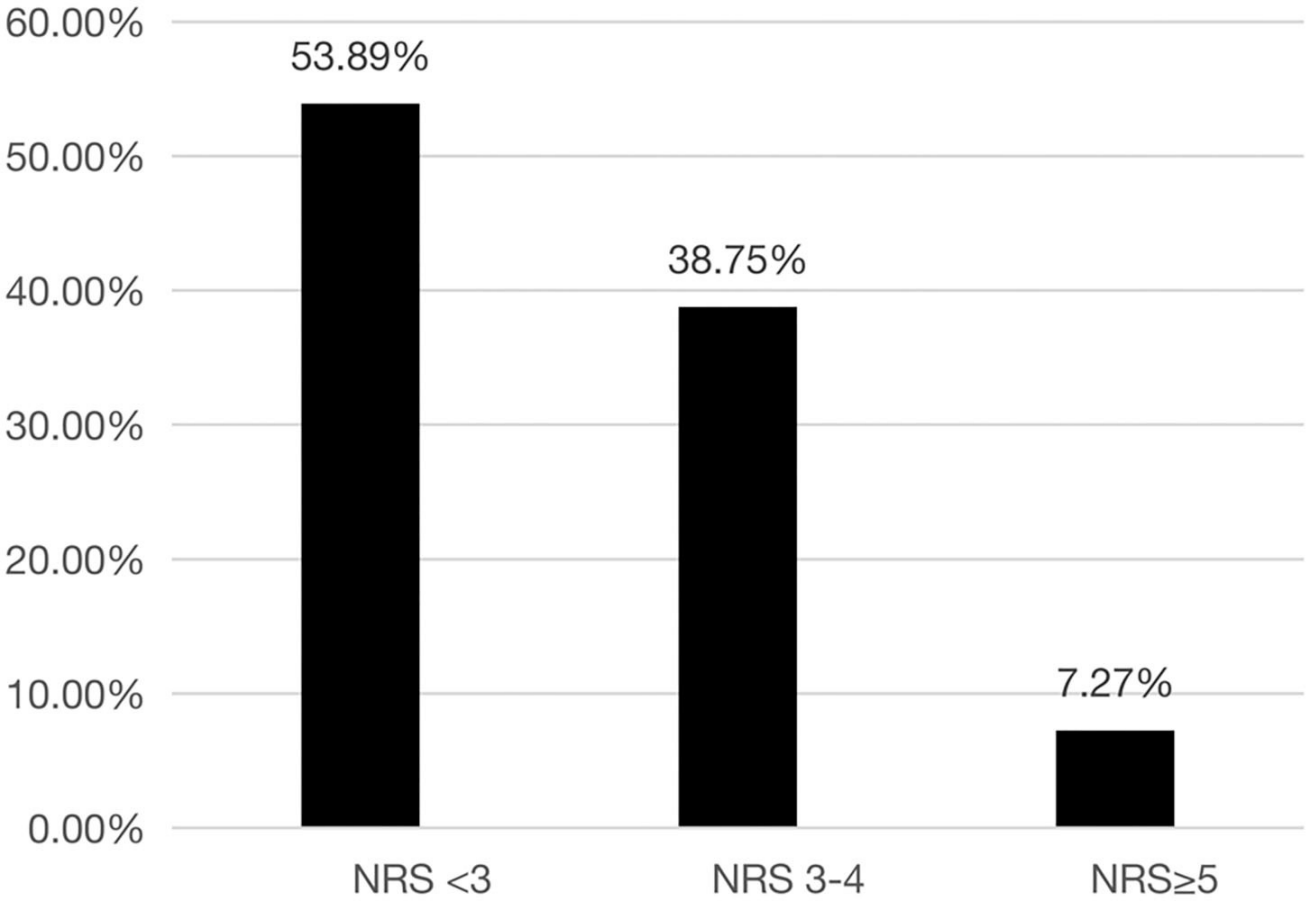
Distribution of different NRS-2002 score stratifications among the patients hospitalized in the Department of Respiratory and Critical Care Medicine of Tibet Autonomous Region People's Hospital. NRS-2002, Nutrition Risk Screening 2002.

**Figure 2:**
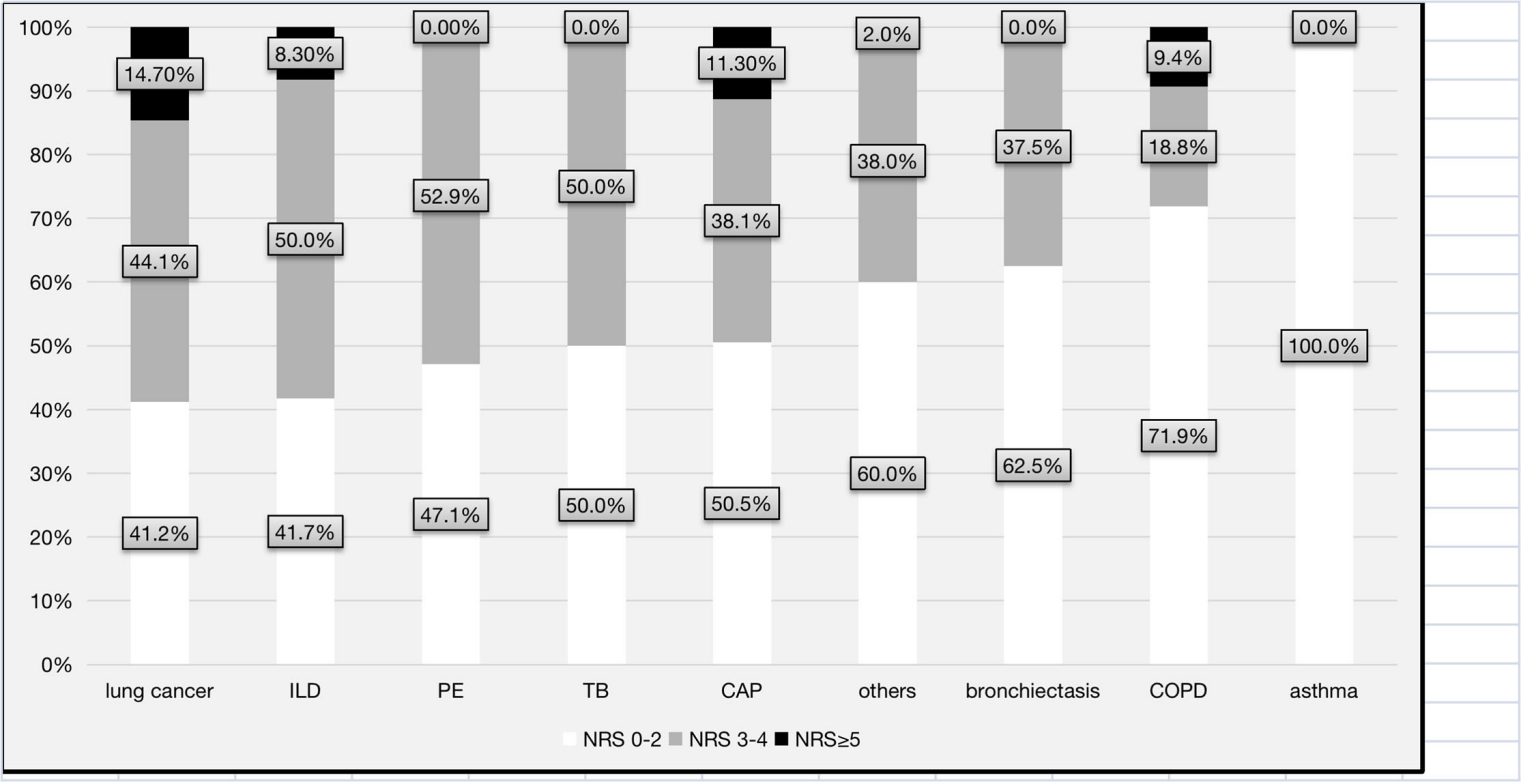
Distribution of the levels of nutritional risk for each major respiratory diagnosis. CAP, community-acquired pneumonia; COPD, chronic obstructive pulmonary disease; TB, tuberculosis or tuberculous pleurisy; PE, pulmonary embolism; ILD, interstitial lung disease; NRS-2002, Nutrition Risk Screening 2002.

Overall, 144 (46.1%) patients were classified as having nutritional risk, with an NRS-2002 score ≥ 3, while 21 (7.3%) were classified as having high nutritional risk, with an NRS-2002 score ≥ 5 (Figure 1). The proportions of patients with NRS-2002 scores of 3–4 or ≥5 among the different major respiratory diagnoses were presented in Figure 2. The prevalence of nutritional risk (NRS-2002 score ≥3) was highest among patients with lung cancer (58.8%), followed by patients with ILD, PE and TB, for whom the prevalence of nutritional risk was ≥50.0%. High nutritional risk (NRS-2002 score ≥5) was more common among patients with lung cancer, CAP, COPD and ILD than among patients with other diagnoses.

## Discussion

The prevalence of nutrition risk is substantial and may vary significantly across different patient populations. Our study is the first to investigate the prevalence of nutritional risk among hospitalized patients permanently living in a very high-altitude region. Our data showed that the prevalence of nutritional risk among patients hospitalized in the department of respiratory care was 46.1%, which was higher than that in a recent pooled analysis study demonstrating that 34.69% of pulmonology inpatients were at nutritional risk (9) in plains areas. A study conducted in Guangzhou (10) showed that the overall prevalence rates of undernutrition and nutritional risks were 17.8 and 41.5%, respectively. The respiratory department had the highest prevalence of undernutrition (28.2%) and nutritional risk (55.9%). The prevalence of nutritional risk was significantly higher among patients ≥ 70 years of age than among patients < 70 years (64.2 vs. 32.6%, *p* < 0.001). No sex difference was observed in the prevalence of nutritional risk in general.

A variety of aspects could contribute to our observations. As mentioned above, the Tibetan Plateau has a unique natural environment located at the highest point of the world. Tibetan residents have a diet characterized by high meat and animal product consumption and low fruit and vegetable consumption. High altitude and hypoxia can influence the metabolism of many nutritional components, including glucose, amino acids, protein and vitamins (18–21). This may be one of the reasons for the high nutritional risk among our patients. Additionally, patients hospitalized in the respiratory department are more likely to be elderly and have more comorbidities than patients in other departments, which may have led to higher NRS-2002 scores. Treatment should be aimed at the etiology and dietary adjustments. For patients who are malnourished, oral nutritional supplements or enteral nutrition may be effective. Additionally, medical staff should pay attention to individuals at high nutritional risk and those with malnutrition, and timely nutritional intervention is essential.

In addition, in our study, nutritional risk varied among patients with different major respiratory diagnoses. As expected, nutritional risk and high nutritional risk were most prevalent (58.8%) among patients with lung cancer in our study. These results were consistent with previous studies showing that ~45.3–65.8% of lung cancer patients in the Czech Republic and Italy were at nutritional risk using the NRS-2002 scoring system (22, 23). Many patients with cancers suffer from loss of appetite and anorexia and increased catabolic metabolism, accounting for their high risk of malnutrition (24). It is suggested that early identification and treatment of malnourished patients or those at increased risk of malnutrition should be routinely performed in the clinic for lung cancer patients, regardless of whether they have a normal or heavy body weight.

Patients with ILD also had a high rate of nutritional risk (58.3%) in our study. Similarly, a recent study showed that nearly one-third (28%) of idiopathic pulmonary fibrosis (IPF) patients in France (25) were malnourished. Several factors, including an increased respiratory muscle load, the release of inflammatory mediators, hypoxemia, reduced food intake due to loss of appetite or loss of muscle mass due to physical inactivity, may negatively affect the nutritional status of ILD patients (26–29). There are two primary mechanisms by which ILD may lead to changes in muscle mass and function. First, ILD frequently leads to inactivity, muscle disuse, and deconditioning as patients attempt to avoid the uncomfortable symptoms of exertional dyspnea. Second, patients with ILD frequently have risk factors for myopathy, including alterations in sex and growth hormone levels (28). Patients with IPF have lower levels of the steroid hormone dehydroepiandrosterone (DHEA) (30), potentially explaining the more consistent and stronger association of ILD severity with unfavorable body composition and low physical function for men than for women. Furthermore, DHEA has antifibrotic effects on human fibroblasts *in vitro*, indicating a need for future studies to explore the links between hormones, nutritional status, and disease progression in patients with fibrotic ILD.

Approximately 50% of our patients with TB were assessed as being at nutritional risk, which was slightly lower than the prevalence reported in previous studies (57.2–64.1%) (11, 27, 31). Patients with TB suffer from decreased appetite and gastrointestinal dysfunction, which leads to insufficient nutrient intake and decreased anabolism (11).

Our data showed that only 28.2% of the COPD patients were at nutritional risk, which was lower than the proportions in previous studies (35.2–54.8%) (13, 32–34). However, we observed that COPD was still the third leading cause of high nutritional risk in our study. The discordant results could be explained by differences in the COPD populations, screening tools, or cutoff criteria (35, 36). Increased resting energy expenditure, systemic inflammation, and chronic hypoxia caused by COPD can lead to further weight loss and malnutrition (37).

The second-highest nutritional risk group in this study was among patients with CAP (11.3%). Poor nutritional status is an established risk factor for CAP (38), and the nutritional status of patients who develop pneumonia often deteriorates before the onset of pneumonia (39). Patients with severe CAP have inflammatory immune activation (manifested by an increased IL-6 level and IL-16/IL-10 ratio), acquired growth hormone (GH) resistance, and insulin resistance (40). The systemic inflammatory response may accelerate catabolism and increase the risk of malnutrition.

Finally, our results showed that several blood biomarkers, including albumin, hemoglobin, CRP levels and red blood cell counts, were significantly different among patients with different nutritional risk score stratifications. As expected, in our study, the serum level of albumin was lower in patients with nutritional risk. It decreased as the nutritional risk increased, while serum CRP levels increased with increasing nutritional risk (41). In addition, the Global Leadership Initiative on Malnutrition (GLIM) guidelines suggest that serum CRP could be used as a surrogate to evaluate the presence and severity of disease/inflammation (42), which contribute to the development of malnutrition (43).

Our study had some of the following limitations. First, it was a single-center, observational stud. However, the area of the Tibet Autonomous Region is more than 1.2 million square kilometers, and other hospitals located in different high-altitude areas were not included in our study. Therefore, the study findings may not be generalizable. The study requires careful interpretation. Second, the sample size was relatively small, and the participants were mainly recruited during the winter and the spring. Different seasons may have different impacts on disease status for patients with respiratory disorders. Third, as patients who were unable to be weighed or measured because of critical illness or who were unable to report food intake or weight loss because of cognitive dysfunction were excluded from our analysis, it is likely that the overall prevalence of nutritional risk was underestimated. Fourth, this survey mainly focused on hospitalized patients. Outpatients with respiratory diseases living at high altitudes were not assessed in our study, and the status of nutrition risk may be different in these patients. In addition, reporting bias due to inaccurate estimations of food intake or weight loss could not be avoided for some patients.

## Conclusion

On the Tibetan Plateau, the nutritional risk was common among patients hospitalized in the respiratory department. Patients with lung cancer, interstitial lung disease, pulmonary embolism, and tuberculosis were more likely to be at nutritional risk. Those with lung cancer, pneumonia and COPD were more likely to be at high nutritional risk. Clinicians should pay more attention to these patients and optimal strategy of nutrition therapy should be implemented to improve their clinical outcomes. To date, evaluation of nutritional status and physical fitness, the nutrition care process, and physical activity are all largely unexplored issues related to the clinical management of patients with lung disease. Further studies will could be performed to assess whether Tibetan patients with respiratory diseases at nutritional risk on the Tibetan Plateau can benefit from nutrition therapy.

## Data Availability Statement

The original contributions presented in the study are included in the article/Supplementary Material, further inquiries can be directed to the corresponding author/s.

## Ethics Statement

The studies involving human participants were reviewed and approved by the Ethics Committee of Tibet Autonomous Region People's Hospital (No. ME-TBHP-21-KJ-056). The patients/participants provided their written informed consent to participate in this study.

## Author Contributions

All authors made substantial contributions to the conception and design, acquisition of data, or analysis and interpretation of data, took part in drafting the article or revising it critically for important intellectual content, agreed to submit it to the current journal, gave final approval for the version to be published, and agreed to be accountable for all aspects of the work.

## Funding

This work was supported by the National Natural Science Foundation of China (No. 81900641) and the Fundamental Research Funds for the Central University; Research Funding of PKU (BMU2021MX020 and BMU2022MX008).

## Conflict of Interest

The authors declare that the research was conducted in the absence of any commercial or financial relationships that could be construed as a potential conflict of interest.

## Publisher's Note

All claims expressed in this article are solely those of the authors and do not necessarily represent those of their affiliated organizations, or those of the publisher, the editors and the reviewers. Any product that may be evaluated in this article, or claim that may be made by its manufacturer, is not guaranteed or endorsed by the publisher.
