# Supplementary material for: High Prevalence of Nutritional Risk Among Pulmonary Patients Living on the Tibetan Plateau
Source: Front Nutr. 2022 May 10;9:872457. doi: 10.3389/fnut.2022.872457 (PMC9127964; doi:10.3389/fnut.2022.872457)
Supplement: Supplementary file 1 [file Table_1.docx]

**Supplemental Table 1 diagnostic criteria for lung disease**

| **disease** | **abbreviation** | **diagnostic criteria** |
| --- | --- | --- |
| acute exacerbation of chronic obstructive pulmonary disease | AECOPD | ^1^Global Initiative for Chronic Obstructive Lung Disease (GOLD). Global Strategy for the Diagnosis, Management and Prevention of Chronic Obstructive Pulmonary Disease: http://www.goldcopd.org (Accessed on February 28, 2022) |
| community-acquired pneumonia | CAP | ^2^Diagnosis and Treatment of Adults with Community-acquired Pneumonia. An Official Clinical Practice Guideline of the American Thoracic Society and Infectious Diseases Society of America |
| pulmonary embolism | PE | ^3^The Task Force for the diagnosis and management of acute pulmonary embolism of the European Society of Cardiology (ESC). 2019 ESC Guidelines for the diagnosis and management of acute pulmonary embolism developed in collaboration with the European Respiratory Society (ERS): The Task Force for the diagnosis and management of acute pulmonary embolism of the European Society of Cardiology (ESC) |
| pulmonary tuberculosis | TB | ^4^Management of Tuberculosis： A Guide to Essential Practice |
| interstitial lung disease | ILD | ^5^Interstitial Lung Disease, 5th ed |
| Asthma | Asthma | ^6^Global Initiative for Asthma (GINA). Global Strategy for Asthma Management and Prevention. www.ginasthma.org (Accessed on August 24, 2021). |

[1]. Global Initiative for Chronic Obstructive Lung Disease (GOLD). Global Strategy for the Diagnosis, Management and Prevention of Chronic Obstructive Pulmonary Disease: http://www.goldcopd.org (Accessed on February 28, 2022).

[2]. Metlay JP, Waterer GW, Long AC, Anzueto A, Brozek J, Crothers K, Cooley LA, Dean NC, Fine MJ, Flanders SA, Griffin MR, Metersky ML, Musher DM, Restrepo MI, Whitney CG. Diagnosis and Treatment of Adults with Community-acquired Pneumonia. An Official Clinical Practice Guideline of the American Thoracic Society and Infectious Diseases Society of America. Am J Respir Crit Care Med. 2019 Oct 1;200(7): e45-e67. doi: 10.1164/rccm.201908-1581ST. PMID: 31573350; PMCID: PMC6812437.

[3]. Konstantinides SV, Meyer G, Becattini C, Bueno H, Geersing GJ, Harjola VP, Huisman MV, Humbert M, Jennings CS, Jiménez D, Kucher N, Lang IM, Lankeit M, Lorusso R, Mazzolai L, Meneveau N, Áinle FN, Prandoni P, Pruszczyk P, Righini M, Torbicki A, Van Belle E, Zamorano JL; The Task Force for the diagnosis and management of acute pulmonary embolism of the European Society of Cardiology (ESC). 2019 ESC Guidelines for the diagnosis and management of acute pulmonary embolism developed in collaboration with the European Respiratory Society (ERS): The Task Force for the diagnosis and management of acute pulmonary embolism of the European Society of Cardiology (ESC). Eur Respir J. 2019 Oct 9;54(3):1901647. doi: 10.1183/13993003.01647-2019. PMID: 31473594.

[4]. Management of Tuberculosis：A Guide to Essential Practice 2019 <https://theunion.org/technical-publications/management-of-tuberculosis-a-guide-to-essential-practice>

[5]. Schwartz M, King Jr TE. Interstitial Lung disease, 5th ed, People's Medical Clearing House, Shelton, CT 2011

[6]. Global Initiative for Asthma (GINA). Global Strategy for Asthma Management and Prevention. www.ginasthma.org (Accessed on August 24,2021
