# Supplementary material for: High Prevalence of Nutritional Risk Among Pulmonary Patients Living on the Tibetan Plateau
Source: Front Nutr. 2022 May 10;9:872457. doi: 10.3389/fnut.2022.872457 (PMC9127964; doi:10.3389/fnut.2022.872457)
Supplement: Supplementary file 2 [file Table_2.docx]

**Supplemental Table 2 Screening for nutritional risk**

| Impaired nutritional status |  | severity of disease (stress metabolism） |  |
| --- | --- | --- | --- |
| Absent | Normal nutritional status | Absent | Normal nutritional requirements |
| Score 0 |  | Score 0 |  |
| Mild | We lost >5% in 3 months | Mild | Hip fracture |
| Score 1 | or | Score 1 | Chronic patients, in particular with  acute complication: cirrhosis，COPD,  Chronic hemodialysis, diabetes,  oncology |
|  | A food intake below 50-75% of the normal requirement in the preceding week |  |  |
| Moderate | Weight loss >5% in 2 months or BMI 18.5-20.5 impaired general condition | Moderate | Major abdominal surgery, stroke  severe pneumonia, hematologic  malignancy |
| Score 2 | Or | Score 2 |  |
|  | A food intake below 25-50% of the normal requirement in the preceding week |  |  |
| Severe | We loss >5% in 1 months（>15% in 3 months） | Severe | Head injury |
| Score 3 | or | Score 3 | Bone marrow transplantation |
|  | A food intake below 0-25%+ of the normal requirement in the preceding week |  | Intensive care patients (APACHE 10) |
| Score: |  |  |  |
| Total score: |  |  |  |
| Calculate the total score: |  |  |  |
|  | 1.Find score (0-3) for Impaired nutritional status (only one: choose the variable with the highest score) and Severity of disease (stress metabolism, i.e., increased nutritional requirements) | | |
|  | 2.Add the two scores (total score) | | |
|  | 3.If age ≥70 years: add 1 to the total score to correct for the frailty of elderly | | |
|  | 4.If age-corrected total ≥3, start nutritional support | | |
